# Metabolic syndrome and physical activity in southern Brazilian community-dwelling elders: a population-based, cross-sectional study

**DOI:** 10.1186/1471-2458-9-25

**Published:** 2009-01-21

**Authors:** Roberta R Dalacorte, César L Reichert, José L Vieira

**Affiliations:** 1 Institute of Geriatrics and Gerontology, Pontifical Catholic University, Porto Alegre, RS, Brazil; 2 Physiology Exercise Laboratory, Feevale University Center, Novo Hamburgo, RS, Brazil; 3 Institute of Cardiology of Rio Grande do Sul, Porto Alegre, Brazil

## Abstract

**Background -:**

The association between a sedentary lifestyle and obesity is well documented, and is linked to an increased prevalence of metabolic syndrome (MS). There is some evidence that information regarding the health benefits of physical activity is beginning to impact on the elderly people and is beginning to change their behavior. We aimed to investigate the level of physical activity undertaken by elderly people with MS and those without this condition.

**Methods -:**

We evaluated 362 community-dwelling elders of Novo Hamburgo, southern Brazil. Diagnosis of MS was based on the International Diabetes Federation criteria and the physical activity (PA) level was estimated by the International Physical Activity Questionnaire. Analysis of covariance was carried out to verify associations between MS risk factors and the level of PA. Logistic regression was used to estimate the MS odds ratio for each level of PA.

**Results -:**

No significant association was found between MS and the level of physical activity, irrespective of sex. The odds ratio for the presence of MS adjusted for sex and age and using insufficiently active elderly people as reference was 1.04 (95% CI, 0.6 to 1.7) in sufficiently active elderly people and 1.15 (95% CI, 0.7 to 2.0) in very active elderly people.

**Conclusion -:**

The elderly citizens of a southern Brazilian community who were diagnosed with MS presented the same levels of PA as the individuals who did not have this diagnosis. This may imply that information on the importance of physical activity has already reached this higher risk population.

## Background

Metabolic syndrome (MS), characterized by central obesity, dyslipidemia, hyperglycemia and hypertension is currently a major global public health challenge because it involves a serious risk of cardiovascular disease and type 2 diabetes [1]. According to the World Health Organization, since 1990 coronary heart disease has been the highest cause of death and this tendency has not yet been observed only in countries with very low life expectancy [2].

It has been reported that technology, automation and a more comfortable lifestyle encourage sedentary behavior, especially in urban populations. The diseases associated with inactivity are now an important global public health problem, with 11.7% of deaths in developed countries being linked to increased obesity and MS [2-4].

The fact that the lack of physical activity and MS are cardiovascular risk factors that increase overall morbidity makes the study of their interrelationships extremely important. This is becoming even more significant considering that the prevalence of physical inactivity, obesity and MS increases with age [5,6] and the world-wide number of elderly people is increasing, especially in newly-industrialized countries such as Brazil [7].

In recent decades, the importance of physical activity for the maintenance of a healthy weight and the prevention of cardiovascular events and death has been widely reported in the scientific literature and the mass media. Clinical studies have been conducted to determine the influence of primary care counseling on the level of physical activity and the maintenance of changes in behavior regarding physical activity. Most of them have shown that recommending physical activity can cause an increase in weekly energy expenditure, even in the elderly [8-10]. However, because of the heterogeneity of these studies, evidence is still insufficient to affirm that primary level counseling is definitely effective in increasing and maintaining the level of physical activity in all ages [11]. To our knowledge, there are not studies showing the impact of the dissemination by the mass-media of information regarding physical activity and how, if at all, such information results in changes of behavior related to physical activity.

As the elderly with MS are at higher risk of cardiovascular disease, they could receive more counseling from their doctors and others health care professionals or also be more suitable to the influence of information available in the media that could lead to changes in their behavior. The hypothesis that we tested in this study was whether elderly people in the community who have been diagnosed with MS are engaged in the same level of physical activity as those without such a diagnosis.

## Methods

We carried out a cross-sectional population-based study with the participation of senior citizens allocated to the second stage of the Longitudinal Study on Aging that was conducted by Feevale University Center in the city of Novo Hamburgo (approximately 236,000 inhabitants, principally of German origin) in the southern Brazilian state of Rio Grande do Sul. The first stage of the study was realized in 2001 by selecting a probabilistic sample of individuals aged between 60 and 79 y (n = 426) calculated at the 95% confidence interval with a 5% margin of error and controlled for age, sex, location in the city and economic class using data from the census conducted in the year 2000 by the Brazilian Institute of Geography and Statistics that revealed about 17.000 people over the age of 60 y living in Novo Hamburgo [12].

For the second stage of the study, data were collected from January to July 2005 when an attempt was made to contact all the participants of the first stage by telephone or letter. Of the 426, 16 had died, according to family members, and 80 were not found. Of the 330 remaining, 173 were willing to participate. We tried to replace the 253 lost participants using the same criteria as that used in the first stage to maintain a probabilistic sample. After 270 new individuals being invited to participate a total of 362 elderly (246 women and 116 men), ranging in age from 60 and 79 y with a mean age of 68 y, agreed to take part in the study. Men were more reluctant to take part in this investigation, as more than 50% of the men invited to this stage of the study declined to participate compared to 10% of women. The project was approved by the ethical committee of São Lucas Hospital, Pontifical Catholic University, RS, Brazil and all participants signed an informed consent form.

Each participant was invited to a clinical assessment during which they answered a short form of the International Physical Activity Questionnaire (IPAQ) [13]. The internationally accepted protocol was used to estimate the weekly calorie expenditure expressed as metabolic equivalents per week (MET/wk) or converted to kcal/wk using the relationship kcal = MET × weight ÷ 60. The participants were classified based on their weekly energy expenditure as follows: ≤ 600 MET/wk, insufficiently active (IA); 601 to 1500 MET/wk, sufficiently active (SA); and ≥ 1500 MET/wk, very active (VA) [14].

Clinical parameters including blood pressure, height, weight, and waist circumference were measured by trained researchers. Weight and height were measured using a mechanical anthropometric scale (Welmy^®^, SP, Brazil) and body mass index (BMI) calculated as the weight in kg divided by the square of the height in meters. For waist, circumference an inelastic tape was used to measure the diameter of the abdomen midway between the lower ribs and iliac crests at the end of expiration and in a standing position. Blood pressure (BP) was taken using a standard mercury sphygmomanometer one time on each arm in subjects who have been lying down for 10 minutes, and the mean of two measurements was used for analyses.

Blood samples were obtained after a 12-hour fast and analyzed in the Biomedicine Laboratory of the Feevale University Center. Labtest^® ^kits were used to assess fasting blood glucose, total cholesterol, high-density lipoprotein-cholesterol (HDL-C) and triglycerides. The samples were analyzed using an enzymatic method.

According to the International Diabetes Federation (IDF) definition for a Europid population subjects were considered to have MS if they had central obesity (defined as waist circumference ≥ 94 cm for men and ≥ 80 cm for women), plus any two of the following four factors: raised fasting plasma glucose ≥ 100 mg/dL (5.6 mmol/L), or previously diagnosed type 2 diabetes; systolic BP ≥ 130 or diastolic BP ≥ 85 mm Hg, or treatment of previously diagnosed hypertension; HDL-C < 40 mg/dL (1.0 mmol/L) in males and < 50 mg/dL (1.3 mmol/L) in females, or specific treatment for this lipid abnormality; TG level ≥ 150 mg/dL (1.7 mmol/L), or specific treatment for this lipid abnormality [1].

Mean and standard deviation (SD) were calculated for parametric data and median plus 25 to 75 percentiles for non-parametric data, with categorical data being represented by number and percentage. The chi-squared test and student *t *test were used for comparison between the MS and without MS groups. Analysis of covariance (ANCOVA) with age-adjusted means was used to evaluate the association between MS component factors and the level of physical activity in both sexes. Logistic regression using a model adjusted for age and sex was used to calculate the odds ratio for MS diagnosis in each physical activity stratum. When appropriate, non-parametric data were log transformed prior to analysis. The significance level was α = 0.05 for a two-tailed test. All statistical analyses were carried out using the SPSS statistical program version 13 (SPSS Chicago, IL).

## Results

Table 1 summarizes the characteristics of the subjects by sex. According to the IDF definition 64% of the women and 44% of the men had MS. High BP was the most frequent finding (84% of the total sample), with 86% of women and 79% of men having a systolic/diastolic pressure ≥ 130/85 mmHg. Systolic pressure was significantly higher (p < 0.001) only in women in the MS group and diastolic pressure was significantly higher (p < 0.001) in both sexes, again only in the MS group. The MS group showed significantly lower (p < 0.001) mean HDL-C values and significantly higher (p < 0.001) glycemia, triglyceride levels and anthropometric values (weight, waist circumference and BMI). See additional file 1: Table 1 for original data about clinical characteristics for men and women in relation to the presence of metabolic syndrome (MS) or its absence (non-MS).

**Table 1:** Clinical characteristics for men and women in relation to the presence of metabolic syndrome (MS) or its absence (non-MS).

	**Men**			**Women**		
		
**Parameters**	**MS** **(n = 51)**	**non-MS** **(n = 65)**	***p***	**MS** **(n = 158)**	**non-MS** **(n = 88)**	***p***
	
Age (years)	68.3 ± 5.3	67.9 ± 5.1	0.63	67.7 ± 4.9	69.0 ± 5.4	0.06
Weight (kg)	83.7 ± 9.6	68.0 ± 10.8	< 0.001 *	73.1 ± 11.7	58.5 ± 9.9	< 0.001 *
Height (cm)	166.5 ± 6.8	166.8 ± 6.5	0.75	154.4 ± 6.4	153.3 ± 5.9	0.19
Body mass index (kg/m^2^)	30.2 ± 2.7	24.5 ± 3.4	< 0.001 *	30.8 ± 4.5	25.0 ± 3.9	< 0.001 *
Waist circumference (cm)	104.5 ± 6.6	89.5 ± 9.3	< 0.001 *	96.0 ± 9.1	81.0 ± 9.2	< 0.001 *
Glucose (mg/dL)	107 ± 52	91 ± 28	0.03 *	101 ± 46	84 ± 15	< 0.001 *
Triglyceride (mg/dL) †	171 (104 to 234)	103 (83 to 129)	< 0.001 *	150 (111 to 210)	109 (87 to 129)	< 0.001 *
HDL-C (mg/dL)	35 ± 10	45 ± 14	< 0.001 *	42 ± 8	50 ± 12	< 0.001 *
Systolic BP (mmHg)	146 ± 25	138 ± 25	0.12	149 ± 21	136 ± 28	< 0.001 *
Diastolic BP (mg/dL)	86 ± 12	82 ± 11	0.05 *	86 ± 12	80 ± 15	0.001 *
Central obesity ‡	51 (100)	16 (25)	< 0.001 *	158 (100)	35 (40)	< 0.001 *
High blood pressure §	48 (94)	44 (68)	< 0.001 *	150 (95)	61 (70)	< 0.001 *
High glycemia ¶	21 (41)	11 (17)	0.004 *	53 (34)	9 (10)	< 0.001 *
High Triglyceride ||	30 (59)	10 (15)	< 0.001 *	78 (50)	12 (14)	< 0.001 *
Low HDL-C * *	37 (72)	24 (37)	< 0.001 *	141 (89)	36 (41)	< 0.001 *

The data represent averages ± SD, number (%) or, for triglycerides, the median value (25th to 75th percentile).

* Significant difference between the MS and non-MS values.

† Data were log transformed prior to analysis.

‡Central obesity was considered as a waist circumference ≥ 80 cm in women and ≥ 94 cm in men.

§High blood pressure was ≥ 130/85 mmHg or use of antihypertensive medication.

¶High glycemia was ≥ 100 mg/dL or diagnosed type 2 diabetes.

||High triglyceride was > 150 mg/dL, fasting.

* * Low high-density lipoprotein cholesterol (HDL-C) was < 40 mg/dL in men and < 50 mg/dL in women, fasting HDL-C, high-density lipoprotein-cholesterol.

Regarding anthropometric values, the waist circumference were ≥ 94 cm in 58% of the men and ≥ 80 cm in 79% of the women, obesity (BMI of 30) occurring in 26% of the men and 36% of the women.

Central obesity was present in 25% of the men and 40% of the women in the non-MS group, but despite this, the difference in relation to the MS group was significant (*p *< 0.001). As expected, the other MS components (high blood pressure, high glycemia, hypertriglyceridemia and low HDL-C) were all significantly more prevalent for both sexes in the MS group.

For mean weekly metabolic energy expenditure expressed as MET/wk there was no significant difference between the MS and non-MS groups for either sex, but there was a significant difference (*p *< 0.001) between MS and non-MS men in mean energy expenditure, that includes the body weight in its formula and is expressed in kcal/wk. There was no significant difference between the MS and non-MS groups regarding activity categories (IA, SA and VA), which were proportionally distributed between the groups. (Table 2)

**Table 2:** Weekly energy expenditure and physical activity level for men and women in relation to the presence of metabolic syndrome (MS) or its absence (non-MS).

	**Men**			**Women**		
		
**Parameters**	**MS** **(n = 51)**	**non-MS** **(n = 65)**	***p***	**MS** **(n = 158)**	**non-MS** **(n = 88)**	***p***
	
**Weekly energy expenditure**						
MET/wk †	1674 ± 232	1374 ± 208	0.34	1496 ± 126	1438 ± 169	0.78
Kcal/wk ‡	2393 ± 298	1557 ± 266	0.04 *	1824 ± 145	1417 ± 195	0.10
						
**Physical activity level (MET/wk)**						
Insufficiently active (< 600)	18 (35)	20 (31)	0.87	50 (32)	33 (38)	0.61
Sufficiently active (601 to 1500)	14 (28)	20 (31)		60 (38)	32 (36)	
Very active (> 1500)	19 (37)	24 (38)		48 (30)	23 (26)	

Values showing dispersion are means ± SD and number (percentage).

* Significant difference between the MS and non-MS values by the chi-squared test at the stated probability level.

^†^Weekly energy expenditure in metabolic equivalents per wk.

‡ Weekly energy expenditure in kilocalories = MET × body mass (Kg) ÷ 60.

No association was found between physical activity and anthropometric measurements such as BMI and waist circumference, although there was a tendency (*p *= 0.05) for the BMI to be higher in SA and VA women compared to IA women. There was no statistically significant association between the level of physical activity and glycemia, triglycerides or HDL-C in either men or women. There was no association between blood pressure and physical activity. See additional file 2: Table 3 for original data about risk factors for metabolic syndrome at different physical activity levels for men and women.

**Table 3:** Risk factors for metabolic syndrome at different physical activity levels for men and women.

	**Men**				**Women**			
	Activity level category *				Activity level category *			
	
**Parameters**	**IA** **(n = 36)**	**SA** **(n = 34)**	**VA** **(n = 43)**	***p***	**IA** **(n = 82)**	**SA** **(n = 91)**	**VA** **(70)**	***p***
Body mass index (kg/m^2^)	26.7 ± 0.7	26.3 ± 0.7	27.8 ± 0.6	0.29	27.7 ± 0.6	29.7 ± 0.5	28.5 ± 0.6	0.053
Waist circumference (cm)	96.0 ± 1.9	94.8 ± 1.9	97.2 ± 1.7	0.63	89.5 ± 1.3	91.6 ± 1.2	90.6 ± 1.4	0.49
Glucose (mg/dL)	98 ± 7	102 ± 7	93 ± 6	0.58	92 ± 4	96 ± 4	97 ± 5	0.77
Triglycerides (mg/dL)^†^	129	115	127	0.64	133	138	141	0.77
HDL-C (mg/dL)	41 ± 2	40 ± 2	41 ± 2	0.99	44 ± 1	46 ± 1	44 ± 1	0.46
Systolic BP (mmHg)	136 ± 4	145 ± 4	144 ± 4	0.25	140 ± 3	145 ± 3	147 ± 3	0.29
Diastolic BP (mmHg)	84 ± 2	83 ± 2	84 ± 3	0.87	81 ± 2	85 ± 1	84 ± 2	0.18

Values for the different parameters were adjusted for age and are means ± standard error (SE).

* Measured in metabolic equivalents per wk (MET/wk): IA = insufficiently active (≤ 600 MET/wk); SA = sufficiently active (601 to 1500 MET/wk); and VA = very active (≥ 1500 MET/wk).

^† ^Data were log transformed prior to analysis; SE not available in the model.

BP = blood pressure; HDL-C = high-density lipoprotein-cholesterol.

The table 4 shows the results for the logistic regression of the MS on physical activity levels (IA, SA and VA), adjusted for age and sex. The odds of having MS was the same for all three physical activity categories, even when the data was separated into pre-defined age bands.

**Table 4:** Odds ratios of having metabolic syndrome at different physical activity levels.

	Activity level*		
	
**Group**	**IA** **(n = 121)**	**SA** **(n = 126)**	**VA** **(n = 114)**
**All individuals**			
Individuals with MS, number (%)	68 (56)	74 (59)	67 (59)
Odds ratio † (95% CI)	1.0	1.04 (0.6 – 1.7)	1.15 (0.7 – 2.0)
			
**Individuals aged 60–69 years**			
Individuals with MS, number (%)	38 (56)	53 (60)	46 (61)
Odds ratio ‡ (95% CI)	1.0	1.15 (0.6 – 2.2)	1.37 (0.7 – 2.7)
			
**Individuals aged 70–79 years**			
Individuals with MS, number (%)	30 (57)	21 (55)	21 (55)
Odds ratio ‡ (95% CI)	1.0	0.94 (0.4 – 2.2)	0.95 (0.4 – 2.2)

* Measured in metabolic equivalents per wk (MET/wk): IA = insufficiently active (≤ 600 MET/wk); SA = sufficiently active (601 to 1500 MET/wk); and VA = very active (≥ 1500 MET/wk).

† Adjusted for age and sex.

‡ Adjusted for sex.

The IA column gives the reference odds ratio.

No difference was found in duration of walking, moderate and vigorous physical activity between MS and non-MS groups for men and women. (Table 5)

**Table 5:** Weekly physical activity duration (min/wk) for men and women in relation to the presence of metabolic syndrome (MS) or its absence (non-MS).

	**Men**			**Women**		
		
**Physical activity**	**MS** **(n = 51)**	**non-MS** **(n = 65)**	***p***	**MS** **(n = 158)**	**non-MS** **(n = 88)**	***p***
	
Walking	216 ± 39	168 ± 34	0.35	127 ± 13	131 ± 18	0.86
Moderate	183 ± 43	182 ± 38	0.98	249 ± 27	232 ± 37	0.72
Vigorous	28 ± 10	8 ± 9	0.15	10 ± 3	9 ± 4	0.81

Values for the different parameters were adjusted for age and are means ± standard error (SE).

## Discussion and conclusion

These findings showed that, in the senior population studied and irrespective of sex, individuals with MS presented the same level of physical activity as those without MS. In spite of the small sample size, the selection procedure was adequate to give 95% confidence interval of ± 5% in the prevalence of MS and a satisfactory statistical power for detecting differences in weekly energy expenditure. The sample provided a statistical power of more then 95% to detect a difference of 30 MET/week in the MS and non-MS groups in both genders. Our results were similar to the only Brazilian study [15] that has assessed these parameters, albeit with a different population (middle-aged Brazilians of Japanese descent) and different methods for assessing the presence of MS and levels of physical activity.

Previous studies suggest that we should expect a higher number of insufficiently active individuals in the MS group [16,17]. However, MS related disorders take a long time to be established, and it is possible that in this study a large number of the individuals with MS may have been sufficiently active and very active recently, which can potentially be characterized as a selection bias. Another selection bias that must be considered is the low participation rate, especially for men.

Probably the increasing amount of information currently available on the importance of physical activity could have driven individuals with unfavorable clinical conditions (e.g., diabetes, dyslipidemia, hypertension and obesity) to practice more physical activity. The majority of our sample consisted of women. There was a tendency in this study for the BMI to be higher in active women in relation to insufficiently active women. This fact, allied to reports that Brazilian women over 60 are the largest group in terms of medical consultations [18], lead us to believe that the advice given to the women by health-care professionals and the media may have somehow modified the activity profile of our sample.

In these findings, each physical activity category contained about one-third of the participants and the odds associated with having MS was the same in the three categories, which is at variance with a previously published report [16].

It is also possible that the IPAQ could have failed to classify the individuals into the correct physical activity levels. Measurement of habitual physical activity is imprecise, both due the imprecision of physical activity measured by questionnaires and due to normal variability in physical activity levels over time. IPAQ may be too imprecise and the sample size too small to detect difference in physical activity. Some studies have pointed out that one main problem with the IPAQ questionnaire is the tendency to overestimate the level of physical activity in sedentary individuals. It has also been reported that this can be overcome by a careful application of the questionnaire, what occurred in our study [19,20]. We also took care to use only physical activity data pertaining to the seven days prior to the interview because we assumed that when assessing individuals older than 60 years of age this is the better way for preventing recall bias. The multi-centered validation study of IPAQ showed that the reliability of the information regarding the seven days prior to interview was comparable to that obtained concerning a usual week of activity [13].

Although the consensus in the published literature is that lifestyle is an important factor in the genesis and treatment of several of the alterations that constitute MS [19,20], data is still scarce regarding the evaluation of the level of physical activity in individuals of 60 years of age and over with MS.

Men were reluctant to take part in our investigation, a factor already reported in other Brazilian studies and which could be caused by cultural aspects of the population [21,22]. This factor could be a limitation of our study because the sample was 32% men and 68% women and, as mentioned above, Brazilian women over 60 years old are more prone to seek medical advice than Brazilian men and for this reason they could have received more guidance on the importance of physical activity [18].

The prevalence of MS in this study was higher than that reported for Australians over 70 years old with the IDF criteria [23]. In relation to other ethnic groups, the prevalence of MS in our sample was higher than in a Chinese sample of men and women aged between 60 and 95 years old [24]. The prevalence of MS in men was similar to that in the general North-American population and in 35 to 65-year old Mexican men but was lower than that found in 55 to 74-year old German men [25-27]. Our findings showed a high prevalence of MS especially in women for which it was higher than in recent reports using the same IDF criteria [25,26]. The high prevalence of MS in the women in our study may have been because of the rigorousness given to the abdominal waist component in the IDF method, which lowers the cut points. Current studies have shown a higher prevalence of MS in different populations when the IDF criteria are used in comparison to the criteria of the National Cholesterol Education Program: Adult Treatment Panel III (ATP III), but this data is not specific for the over 60 year olds [25,28,29]. The very high prevalence of MS in women in our study is worrying, because the association between this syndrome and cardiovascular events has been described not only among young women with a high risk of coronary disease [30] but also among women of 55 years and older [31,32]. The impact of MS on a sample of Brazilian women over the age of 60 with a low prevalence of cardiovascular disease has also been shown. A prospective study reported that MS was associated with a higher occurrence of cardiovascular events in this population [33].

The mean level of physical activity of our population was 1,485 MET/wk (1,758 kcal/wk) and was lower than that found in other studies, like the mean of 2,514 MET/wk reported in the IPAQ international validation study, although in this case the data refers to a general population sample and not a population of over 60 year olds [13]. A South Africans study using the IPAQ and the Yale Physical Activity Survey to assess the level of physical activity in people over the age of 60 reported a weekly IPAQ energy expenditure very much higher than that of our study [6]. The higher energy expenditure of the individuals in this study could be explained by the sampling methodology used, which selected elderlies who were members of 'lunch clubs' – a factor which could have chosen more active individuals. In our study, the weekly caloric expenditure, expressed in Kcal/week, remained below the minimum of 2000 Kcal/wk recommended for the reduction of morbimortality [34]. Walking is the most common form of leisure-time physical activity [35]. The analysis of the duration of different kinds of physical activity were performed and we didn't find differences in duration of walking, moderate and vigorous physical activity between participants with or without metabolic syndrome, for men and women. There was a tendency for men with MS to have more time of vigorous exercise, but this was not significant. These results were not consistent with the previous studies which showed that the metabolic syndrome was inversely associated with participation and duration in physical activity [5,36].

In summary, in the sample population over 60 year old the level of physical activity was equal in individuals with and without MS. Our hypothesis is that individuals in the sample were aware of the importance of physical activity, and this could suggest that the level of physical activity was equal in both the MS and non-MS due to information and advice regarding the importance of physical activity. Other studies of over 60 years old are needed to make conclusions on causality.

## Competing interests

The authors declare that they have no competing interests.

## Authors' contributions

RRD performed the analysis and interpretation of data and wrote a substantial portion of the manuscript. CR participated in the conception and design of the study and performed the acquisition of data. JLV carried out the statistical analysis and has been involved in revising the manuscript. All authors read and approved the final manuscript.

## Pre-publication history

The pre-publication history for this paper can be accessed here:



## References

[B1] Alberti KG, Zimmet P, Shaw J (2005). The metabolic syndrome–a new worldwide definition. Lancet.

[B2] Murray CJ, Lopez AD (1997). Alternative projections of mortality and disability by cause 1990–2020: Global Burden of Disease Study. Lancet.

[B3] Eckel RH, Grundy SM, Zimmet PZ (2005). The metabolic syndrome. Lancet.

[B4] Yusuf S, Hawken S, Ounpuu S, Dans T, Avezum A, Lanas F, McQueen M, Budaj A, Pais P, Varigos J, Lisheng L (2004). Effect of potentially modifiable risk factors associated with myocardial infarction in 52 countries (the INTERHEART study): case-control study. Lancet.

[B5] Ford ES, Kohl HW, Mokdad AH, Ajani UA (2005). Sedentary behavior, physical activity, and the metabolic syndrome among U.S. adults. Obes Res.

[B6] Kolbe-Alexander TL, Lambert EV, Harkins JB, Ekelund U (2006). Comparison of two methods of measuring physical activity in South African older adults. J Aging Phys Act.

[B7] Ramos R, Rosa TE, Oliveira ZM, Medina MC, Santos FR (1993). [Profile of the elderly in a metropolitan area of southeastern Brazil: results of a domiciliary survey]. Rev Saude Publica.

[B8] Kerse NM, Flicker L, Jolley D, Arroll B, Young D (1999). Improving the health behaviours of elderly people: randomised controlled trial of a general practice education programme. Bmj.

[B9] Steptoe A, Doherty S, Rink E, Kerry S, Kendrick T, Hilton S (1999). Behavioural counselling in general practice for the promotion of healthy behaviour among adults at increased risk of coronary heart disease: randomised trial. Bmj.

[B10] Pinto BM, Goldstein MG, Ashba J, Sciamanna CN, Jette A (2005). Randomized controlled trial of physical activity counseling for older primary care patients. Am J Prev Med.

[B11] U.S. Preventive Services Task Force (2002). Behavioral counseling in primary care to promote physical activity: recommendation and rationale. Ann Intern Med.

[B12] Censo 2000 com divisão territorial 2001. http://www.ibge.gov.br.

[B13] Craig CL, Marshall AL, Sjostrom M, Bauman AE, Booth ML, Ainsworth BE, Pratt M, Ekelund U, Yngve A, Sallis JF, Oja P (2003). International physical activity questionnaire: 12-country reliability and validity. Med Sci Sports Exerc.

[B14] International Physical Activity Questionnaire. http://www.ipaq.ki.se.

[B15] Doro AR, Gimeno SG, Hirai AT, Franco LJ, Ferreira SR (2006). [Analysis on the association of physical activity with metabolic syndrome in a population-based study of Japanese-Brazilians]. Arq Bras Endocrinol Metabol.

[B16] Irwin ML, Ainsworth BE, Mayer-Davis EJ, Addy CL, Pate RR, Durstine JL (2002). Physical activity and the metabolic syndrome in a tri-ethnic sample of women. Obes Res.

[B17] Rennie KL, McCarthy N, Yazdgerdi S, Marmot M, Brunner E (2003). Association of the metabolic syndrome with both vigorous and moderate physical activity. Int J Epidemiol.

[B18] Barreto SM, Kalache A, Giatti L (2006). Does health status explain gender dissimilarity in healthcare use among older adults?. Cad Saude Publica.

[B19] Ekelund U, Sepp H, Brage S, Becker W, Jakes R, Hennings M, Wareham NJ (2006). Criterion-related validity of the last 7-day, short form of the International Physical Activity Questionnaire in Swedish adults. Public Health Nutr.

[B20] Fogelholm M, Malmberg J, Suni J, Santtila M, Kyrolainen H, Mantysaari M, Oja P (2006). International Physical Activity Questionnaire: Validity against fitness. Med Sci Sports Exerc.

[B21] Araujo F, Pereira AC, Latorre Mdo R, Krieger JE, Mansur AJ (2004). High-sensitivity C-reactive protein concentration in a healthy Brazilian population. Int J Cardiol.

[B22] Viebig RF, Valero MP, Araujo F, Yamada AT, Mansur AJ (2006). [Cardiovascular health profile of an adult population from the metropolitan region of Sao Paulo]. Arq Bras Cardiol.

[B23] Adams RJ, Appleton S, Wilson DH, Taylor AW, Dal Grande E, Chittleborough C, Gill T, Ruffin R (2005). Population comparison of two clinical approaches to the metabolic syndrome: implications of the new International Diabetes Federation consensus definition. Diabetes Care.

[B24] He Y, Jiang B, Wang J, Feng K, Chang Q, Fan L, Li X, Hu FB (2006). Prevalence of the metabolic syndrome and its relation to cardiovascular disease in an elderly Chinese population. J Am Coll Cardiol.

[B25] Ford ES (2005). Prevalence of the metabolic syndrome defined by the International Diabetes Federation among adults in the U.S. Diabetes Care.

[B26] Rathmann W, Haastert B, Icks A, Giani G, Holle R, Koenig W, Lowel H, Meisinger C (2006). Prevalence of the metabolic syndrome in the elderly population according to IDF, WHO, and NCEP definitions and associations with C-reactive protein: the KORA Survey 2000. Diabetes Care.

[B27] Ramirez-Vargas E, Arnaud-Vinas MD, Delisle H (2007). Prevalence of the metabolic syndrome and associated lifestyles in adult males from Oaxaca, Mexico. Salud Publica Mex.

[B28] Assmann G, Guerra R, Fox G, Cullen P, Schulte H, Willett D, Grundy SM (2007). Harmonizing the Definition of the Metabolic Syndrome: Comparison of the Criteria of the Adult Treatment Panel III and the International Diabetes Federation in United States American and European Populations. Am J Cardiol.

[B29] Gonzalez-Ortiz M, Martinez-Abundis E, Jacques-Camarena O, Hernandez-Gonzalez SO, Valera-Gonzalez IG, Ramos-Zavala MG (2006). Prevalence of metabolic syndrome in adults with excess of adiposity: comparison of the Adult Treatment Panel III criteria with the International Diabetes Federation definition. Acta Diabetol.

[B30] Kip KE, Marroquin OC, Kelley DE, Johnson BD, Kelsey SF, Shaw LJ, Rogers WJ, Reis SE (2004). Clinical importance of obesity versus the metabolic syndrome in cardiovascular risk in women: a report from the Women's Ischemia Syndrome Evaluation (WISE) study. Circulation.

[B31] Lawlor DA, Ebrahim S, Davey Smith G (2004). The metabolic syndrome and coronary heart disease in older women: findings from the British Women's Heart and Health Study. Diabet Med.

[B32] Lorenzo C, Williams K, Hunt KJ, Haffner SM (2007). The National Cholesterol Education Program – Adult Treatment Panel III, International Diabetes Federation, and World Health Organization definitions of the metabolic syndrome as predictors of incident cardiovascular disease and diabetes. Diabetes Care.

[B33] Cabrera MA, Gebara OC, Diament J, Nussbacher A, Rosano G, Wajngarten M (2007). Metabolic syndrome, abdominal obesity, and cardiovascular risk in elderly women. Int J Cardiol.

[B34] Paffenbarger RS, Hyde RT, Wing AL, Hsieh CC (1986). Physical activity, all-cause mortality, and longevity of college alumni. N Engl J Med.

[B35] Tanasescu M, Leitzmann MF, Rimm EB, Willett WC, Stampfer MJ, Hu FB (2002). Exercise type and intensity in relation to coronary heart disease in men. Jama.

[B36] Bertrais S, Beyeme-Ondoua JP, Czernichow S, Galan P, Hercberg S, Oppert JM (2005). Sedentary behaviors, physical activity, and metabolic syndrome in middle-aged French subjects. Obes Res.

